# Youth Suicide and Self-Harm: Latent Class Profiles of Adversity and the Moderating Roles of Perceived Support and Sense of Safety

**DOI:** 10.1007/s10964-023-01762-1

**Published:** 2023-03-24

**Authors:** Charlotte Silke, Bernadine Brady, Carmel Devaney, Cliodhna O’Brien, Micheal Durcan, Brendan Bunting, Caroline Heary

**Affiliations:** 1 UNESCO Child & Family Research Centre, University of Galway, Galway, Ireland; 2 HSE National Office of Suicide Prevention, Dublin, Ireland; 3 Western Region Drugs & Alcohol Task Force, Galway, Ireland; 4 Ulster University, Coleraine, Northern Ireland UK; 5 School of Psychology, University of Galway, Galway, Ireland

**Keywords:** Suicidality, Adolescents, Risk Profiles, Protective Factors, Social-ecology

## Abstract

Research suggests that exposure to adversity can lead to an increased risk of experiencing suicidal and self-injurious thoughts or behaviours, but few studies have examined whether different patterns of adversity are differentially associated with youth suicide/self-harm. The current study aims to explore the relationship between exposure to adversity across various social domains and youth self-harm and suicidality, using a person centred approach, and examines whether access to social support and a sense of safety across home, peer or school settings buffer the relationship between adversity and self-harm/suicidality. Secondary data analyses were carried out on cross-sectional self-report data collected from 4848 (M_age_=15.78, SD = 0.59; 50% female) adolescents who participated in the Irish Planet Youth survey. Latent Class Analyses identified four distinct profiles of adversity; low-adversity (*n* = 2043, 42%); peer-adversity (*n* = 972, 20%); parental-adversity (*n* = 1189, 25%); and multiple-adversity (*n* = 644, 13%). Findings from logistic moderated regressions indicated that there were significant differences in self-harm and suicidality across the adversity classes. Although parental support and perceived safety at school were negatively associated with suicidality and self-harm outcomes, no significant moderation effects were observed. These findings suggest that youth who experience adversity across multiple social domains are more likely to report suicidal and self-harm thoughts and behaviours, and should be key targets for intervention/prevention efforts. While parental support and school safety may act as significant compensatory factors, further work is needed to identify the social resources that can offset the risk imposed by youth’s adverse experiences.

## Introduction

Research indicates that adolescents are at increased risk of engaging in self-harm and suicidal behaviours (King et al., 2020), and are specified as priority targets for intervention and prevention efforts in both national (Health Service Executive 2020) and international (World Health Organisation; WHO 2021) policy. It is widely contended that targeted, community-focused initiatives are needed to help combat youth engagement in suicide/self-harm, but in order to be effective, these initiatives need to be informed by an understanding of the associated risk and protective factors. Although research (Lopez et al., 2021) and theory (Bronfenbrenner, 1979) indicate that adolescents’ health and development are impacted by their experiences across multiple social-ecological systems, few studies have examined how peer, parent, and school-based risk and protective factors interact to influence youth suicidality/self-harm. The current research aims to address this research gap and generate further insight into the association between youth self-harm/suicidality and the pattern of adversity they experience across multiple social contexts. The research also sets out to examine whether ecological resources, such as social support and sense of safety, help buffer the relationship between adversity and self-harm/suicidality in young people.

Youth suicidality (Liu, Walsh, Sheehan, Cheek & Sanzari, 2022) and self-harm (Rodríguez-Blanco et al., 2021) are regarded as major public health concerns. Recent prevalence estimates suggest that despite heightened public awareness and policy focus in this area, rates of deliberate self-injury, suicidal thoughts, and suicide attempts among adolescents continue to soar (King et al., 2020; McManus et al., 2019). Findings from meta-analyses have indicated that approximately 23% of adolescents engage in deliberate acts of self-harm (Gillies et al., 2018), and between 14–18% of adolescents experience suicidal ideation or thoughts (Nock et al., 2013). Other reports with national samples in the US have indicated that between 6–8% of adolescents attempt suicide (Lim et al., 2019). In Europe, suicide is the second leading cause of death among young people aged 15–19 years (UNICEF, 2021). As adolescence is a key development period, which often marks the onset for self-harm and suicidal thoughts, it is an important stage for intervention and prevention (Robinson, Garisch, & Wilson, 2021). Being able to identify and screen youth who are at elevated risk of experiencing suicidal or self-injurious thoughts/behaviours, and understanding the factors which contribute to this risk, are key to the development of effective intervention and prevention strategies (Asarnow & Mehlum, 2019; Bilsen, 2018), and are cited as important policy objectives (HSE, 2020).

Findings from various systematic reviews and meta-analyses have identified several risk factors associated with self-harm (Abdelraheem, McAloon & Shand, 2019) and suicidality (Cluver, Orkin, Boyes & Sherr, 2015; Flores, Swartz, Stuart & Wilcox, 2020), and indicate that these are complex and dynamic phenomena, influenced by the interplay between numerous biological, psychological, social, and environmental factors (Wasserman et al., 2021). However, some evidence suggests that social or environmental factors may have a stronger association with suicidality than genetic or biological factors (Bruffaerts et al., 2010). A growing body of literature suggests that childhood adversity, or exposure to negative life events, is strongly associated with increased risk of suicidality (Aytur et al., 2022; Li et al., 2021) and self-harm (Russell et al., 2019) among adolescents. For example, previous research indicates that exposure to any form of adverse childhood experience increases the risk of attempting suicide 2-to-5 fold (Dube et al., 2001). Although estimates can vary widely, reports typically suggest that between 50–80% of adolescents are exposed to at least one adverse experience (Broekhof et al., 2022; Lee, Kim & Terry, 2020). The high prevalence of lived adversity during childhood/adolescence, and the strong observed links between negative life events and suicidality and self-harm, make this a priority area for preventative research (Corcoran, Gallagher, Keeley, Arensman & Perry, 2006; Moore & N Ramirez, 2016).

While the number of studies purporting an association between adversity and suicidality/self-harm have proliferated in recent years, understanding about the nature of this relationship has remained somewhat limited, due to a variety of reasons (Göbel & Cohrdes, 2021). First, much of the research in this area has relied on a narrow definition of adversity (Afifi et al., 2020), often focusing on assessments of abuse, maltreatment or household dysfunction (SmithBattle et al., 2021). However, increasing evidence suggests that the relationship between youth adversity and health outcomes is stronger when additional experiences, such as peer victimisation, community violence, or school problems, are included as indicators of adversity (Finkelhor et al., 2013; Wang, Yuan, Chang, Li & Su, 2022). Hence, researchers now propose that our understanding of youth suicidality and self-harm, and our ability to develop targeted interventions, could be improved if a more diverse definition of adversity was utilised (Lacey & Minnis, 2020). In particular, it is argued that, in addition to the traditional assessments of family adversity and abuse, indicators of peer victimisation and school adversity should be included in future measurements of adversity (Wang et al., 2022). Support for this expanded approach to the definition of adversity is provided by the wider theoretical and research base which suggests that youth wellbeing is impacted by the social interactions and experiences they have across multiple ecological contexts (Bearman & Moody, 2004; Lanza, Rhoades, Nix & Greenberg, 2010). For example, according to Bronfenbrenner’s socio-ecological model youth development is shaped by both their immediate environment (e.g. parent and peer relations) and their wider community (e.g. school environment) (Bronfenbrenner, 1979).

Furthermore, research exploring the link between adversity and suicidality/self-harm has traditionally employed a cumulative risk approach (Lee et al., 2020). While the cumulative risk model has some notable advantages, in that it can be easily replicated in research or used as a practical tool for assessing risk in clinical settings (Merians, Baker, Frazier & Lust, 2019), reliance on the cumulative approach as a research or screening tool has recently been criticised (Lacey & Minnis, 2020) for assuming that all adversities influence outcomes equally (Hagan, Sulik & Lieberman, 2016), and for failing to consider the multidimensional nature of adversity (Barboza, 2018; Lee et al., 2020). This may be an important oversight as several studies have indicated that different forms of adversity show different associations with youth health outcomes (Bevilacqua et al., 2021; Li et al., 2021), with some evidence suggesting that youth may be less able to cope when exposed to adversity across multiple social domains (Göbel & Cohrdes, 2021). Understanding whether different clusters or patterns of adversity are more or less strongly associated with youth suicidality and self-harm is important for informing the development of targeted, youth-specific prevention strategies and informing the design of more sophisticated screening tools for at-risk youth. It is argued that person-centered approaches may address some of the issues associated with the cumulative risk model (Lanza et al., 2010), and could help provide unique insights in this area by expanding knowledge about the dominant patterns of adversity experienced by adolescents and increasing our ability to detect which adolescents are most at risk of suicide or self-harm.

Although the importance of identifying predictors of youth suicide and self-harm is widely acknowledged in policy and research, understanding the factors that can help youth adapt or cope when exposed to adversity is similarly important from an intervention and prevention perspective. While protective factors can occur at both the individual and contextual level (Gallagher & Miller, 2018), identifying the social or contextual mechanisms that can offset risk may have more applied relevance, as these factors are often considered more amenable to intervention/prevention strategies (Standley, 2020). Notably, current mental health and suicide prevention policies (e.g., WHO Comprehensive Mental Health Action Plan 2013–2030; HSE Connecting for Life 2015–2024) endorse community-focused initiatives, and emphasise the importance of educating and empowering local communities to prevent and respond to suicide/self-harm behaviour (Rochford et al., 2021). Research and theory supports this policy approach and shows that prevention strategies that aim to promote families, communities or other gatekeepers’ understanding about social protective factors can help reduce youth self-harm and suicide mortality (Asarnow & Mehlum, 2019). Although previous research has identified positive social connections with others, such as social support (Miller, Wakefield & Sani, 2015; Von Cheong, Sinnott, Dahly & Kearney, 2017) and perceived sense of safety (Gallagher & Miller, 2018), as important ecological resources that can help promote wellbeing, more understanding about how these factors buffer the relationship between adversity and youth suicidality/self-harm is needed. This information is crucial for enhancing the development of targeted, community-focused youth prevention strategies.

## Current Study

Youth are cited as a priority target for suicide/self-harm intervention and prevention strategies, but greater understanding about the factors that place youth at risk, and increased awareness of the ecological resources that can protect or buffer against this risk, are needed to help strengthen current policy and practice efforts. While the risk factors for youth suicide/self-harm are multifarious, previous research has highlighted the benefits of taking an adversity-informed approach to suicide/self-harm screening and intervention. However, despite theory and research indicating that youth development is impacted by their experiences across multiple social-systems, few studies have examined whether different patterns of family, school, or peer-based adversity are linked to youth self-harm and suicidality outcomes. Relatedly, there is little knowledge about whether youths’ experiences of positive social connections (e.g., social support or sense of safety), across various social contexts, can act as effective protective factors for vulnerable or at-risk youth. The current research seeks to address these important knowledge gaps and extend understanding of the relationship between adversity and youth self-harm and suicidality. Specifically, this study aims to identify dominant latent class profiles based on the pattern of adversity experienced by adolescents across home, peer and school settings in Ireland (Aim 1), examine the relationship between the observed latent class profiles and youth suicidality and self-harm, after controlling for known socio-demographic (e.g., gender, sexual orientation) covariates (Aim 2), and determine whether school safety, home safety, teacher relationships, parental support or friend support buffer the relationship between the adversity profiles and youth self-harm/suicidality (Aim 3).

## Method

### Participants and Procedures

In 2018, as part of the Irish Planet Youth initiative, all post-primary schools and education centres (*N* = 90) in three west of Ireland counties were invited to take part in a large, youth lifestyle questionnaire, using a clustered random sampling approach. A 99% response rate from schools and education centres was observed. All students in their fourth year of education at participating schools and education centres were then invited to take part in the Planet Youth (PY) survey. An 80% student response rate was observed, based on informed, parental opt-out consent and student assent. Students in these schools completed pen-and-paper self-report questionnaires assessing numerous aspects of their lives and living conditions. The analyses presented in the current study are based on the responses collected from the 4848 (2404 male, 2417 female, 27 not reported) adolescents who participated in the 2018 Irish Planet Youth questionnaire. All participants were aged between 14–18 years (M_age_ = 15.78, SD = 0.59). Ethical approval for the PY questionnaire was granted by the Royal College of Physicians of Ireland (RCPI). Further information about the Planet Youth survey is available elsewhere (see www.planetyouth.ie).

### Measures

#### Adversity/Negative Life Events

Adolescent exposure to adversity across home, school, and peer settings were assessed using 15 single item variables (e.g., *Have you had a serious argument with your parents*?), from the Negative Life Events (Wills, Vaccaro, & McNamara, 1992), and PY victimisation and bullying (Halldorsdottir et al., 2021) measures. All adversity items were coded as binary variables (0=No, 1=Yes), which assessed whether respondents had experienced the negative life event or not.

#### Self-harm

Participants thoughts about engaging in self-harm were assessed with a single item *(e.g., Over your lifetime, have you thought about harming yourself on purpose?)*. Engagement in self-harm behaviours was assessed using a single item (e.g., *Over your lifetime, have you ever harmed yourself on purpose?*). Responses were coded as 0=No, 1=Yes.

#### Suicidality

Participants suicidal thoughts were assessed using one item *(e.g., In the past week, I thought of completing suicide?)*. Participants experience of attempting suicide was assessed using one single indicator (e.g., *Have you ever made an attempt to complete suicide within the last six months?*). Responses were dummy coded as 0=Never, 1=Yes.

#### Parental Support

Participants’ supportive relationships with their parents were measured using the Perceived Parental Support scale (Thorlindsson, Sigfusdottir, Bernburg, & Halldorsson, 1998). The scale consists of 5 Likert-type items (e.g., *Advice about studies*), where participants rate how difficult it is for them to access various supports from parents on a scale of 1 (Very Difficult) to 4 (Very Easy). Higher scores were reflective of more supportive parent-child relationships.

#### Friend Support

Participants’ supportive relationships with their friends were measured using the Perceived Friend Support scale (Thorlindsson et al., 1998). The scale consists of 5 Likert-type items (e.g., *warmth & care*), where participants rate how difficult it is for them to access various supports from their friends on a scale of 1 (Very Difficult) to 4 (Very Easy). Higher scores were reflective of more supportive peer relationships.

#### Teacher Relationships

Participants’ relationship with their teachers was assessed by a single Likert-type item (e.g., *I get on poorly with teachers in my school*), which ranged from 1 (almost always) to 5 (almost never). Scores were recoded so that higher scores were reflective of more positive student-teacher relationships.

#### Home Safety

Participants’ perceptions of home safety were assessed by a single Likert-type item (e.g., *I feel safe at home*), which ranged from 1 (almost never) to 5 (almost always). Higher scores were reflective of a greater sense of safety at home.

#### School Safety

Participants’ perceptions of school safety were assessed by a single Likert-type item (e.g., *I feel safe at school*), which ranged from 1 (almost never) to 5 (almost always). Higher scores were reflective of a greater sense of safety at school.

#### Socio-Demographic Characteristics

Information about participants’ gender (Male=0, Female=1), sexual orientation (No attraction to same sex=0; Some attraction to same sex=1), place of birth (Ireland=0; Other=1), maternal education (Secondary or Lower=0; Technical College=1; University=2) and age (birth year) were collected using observed, single-item variables.

### Data Analysis Plan

The current research involves a secondary data analysis of the Planet Youth dataset. Latent Class Analysis (LCA) was used to examine whether homogenous sub-groups or classes of adolescents could be identified based on their adverse experiences across peer, school and home settings. In order to identify the dominant adversity profiles, a series of LCA models with an ascending number of classes were specified in successive order. Following guidelines specified by Geisser (2013), model selection was based on theory and a comparison of multiple relative fit indices. For each model the Akaike information criterion (AIC), Bayesian information criterion (BIC), and the sample size-adjusted Bayesian criterion (aBIC) were reviewed, where lower values were considered indicative of superior model fit (Nylund, Asparouhov & Muthén, 2007). The Lo-Mendell-Rubin Likelihood Ratio Test (LRT) was used to compare models with different classes, where a significant value indicates support for the k class model over the k-1 model (Weller, Bowen & Faubert, 2020). Entropy, which is a diagnostic statistic that assesses how accurately the model defines classes, was also reviewed for each model, where values closer to 1 are considered indicative of more accurate classification (Weller et al., 2020). Participants’ chance of belonging to each class was determined based on their raw posterior probability scores. Participants were assigned to their “most-likely” class based on these scores. In order to explore interactions between the observed latent classes and moderator variables a series of mixed-effects (moderated logistic regression) models were specified. Separate models were specified for each of the four outcome variables. Socio-demographic variables (e.g., gender, sexual orientation) were included as a covariate in each model, as previous research has indicated that these may be important correlates of self-harm/suicidality (Bresin & Schoenleber, 2015; Craig et al., 2020). All moderators were mean-centred and significant interactions were probed using the pick-a-point (+/− 1SD) approach (Hayes, 2018). As data were collected from adolescents nested within schools, participants’ schools were entered as a cluster variable within all analyses using the Cluster Mixture command, which adjusts the standard errors and fit indices for clustering. All latent class and logistic regression analyses were carried out using mPlus version 8 (Muthén & Muthén, 2017) software. Descriptive analyses, including correlations and chi-square tests, were carried out using SPSS (version 27; IBM, 2022).

### Missing Data

For all analyses, full information maximum likelihood (FIML) was used to handle missing data. Only participants with missing data on all variables or all x-variables (*n* = 211) were excluded from the analyses. FIML is recognised as a superior approach for handling missing data as it is proposed to produce more accurate estimates and standard errors than other approaches, such as listwise/pairwise deletion or single imputation (Lang & Little, 2018). In the current study, the overall level of missingness across all variables was approximately 1%.

## Results

### Descriptive Statistics

Descriptive statistics showed that the most common forms of adversity experienced by young people in this study were having serious arguments with their parent/guardian (*n* = 2240; 46%) and receiving nasty messages from others (*n* = 2233; 46%). Experiencing sexual violence was the adverse event reported by the fewest number (*n* = 153; 3%) of participants (see Table 1).

**Table 1:** Number (Percentage) of Participants Experiencing Each Form of Adversity

List of Adversity Indicators	Yes
1. Experienced Parental Separation or Divorce	903 (19%)
2. Had a serious Argument with your Parents	2240 (46%)
3. Witnessed Your Parents Having Serious Arguments	1934 (40%)
4. Witnessed Psychological Abuse/Violence at Home	877 (18%)
5. Been Involved in Physical Violence at Home	577 (12%)
6. Lost a Parent/Sibling (Death)	408 (8%)
7. Lost a Friend (Death)	873 (18%)
8. Had A Parent Lose their Job	833 (17%)
9. Experienced a Break-Up	1754 (36%)
10. Been Rejected by your Friends	1997 (41%)
11. Received Nasty Messages from Others	2233 (46%)
12. Been Dismissed from Class/Sent to Principal	1326 (27%)
13. Been Teased by a group	2033 (43%)
14. Been physically attacked by a group	525 (11%)
15. Been the Victim of Sexual Violence (in last 12 months)	153 (3%)

Approximately 47% (*n* = 2269) of participants indicated that they had thought about engaging in self-harm, and 33% (*n* = 1605) indicated that they had engaged in self-harm at least once over their lifetime. Additionally, frequency estimates indicated that approximately 20% (*n* = 991) of participants had thought about completing suicide, while 4% (*n* = 210) had attempted suicide in the last six months. Descriptive statistics for all moderating variables are displayed in Table 2. As can be seen here, participants reported moderate-high scores across all moderators.

**Table 2:** Descriptive Statistics for Moderating Variables

	M	SD	Range	α	S	K
Teacher Relations	4.35	0.98	1–5	–	−1.61	2.06
Safety at School	4.36	0.89	1–5	–	−1.50	2.04
Safety at Home	4.79	0.58	1–5	–	−3.46	3.99
Parental Support	16.24	3.34	1–20	0.86	−0.89	0.36
Friend Support	15.95	3.15	1–20	0.83	−0.66	0.28

### Latent Class Analysis (LCA)

An exploratory LCA process was conducted to identify underlying latent classes or profiles of youth based on their responses across a set of 15 binary adversity indicators. A total of 6 LCA models, ranging from 1 to 6 classes, were specified and assessed according to the criteria outlined in the analytic plan. After examining both the fit indices and the theoretical meaning of each model, it was determined that the four-class solution provided the best overall fit. As can be seen in Table 3, the four class solution showed lower AIC, BIC, and aBIC values in comparison to the one, two and three class models and evidenced superior support in comparison to the k-1 models. For the five and six class models improvements in fit indices were miniscule and the LRT value was found to be non-significant, indicating that there was no statistical support for the five or six class model over the four class model. Thus, the four class model was selected as the best fitting model.

**Table 3:** Fit Indices for Latent Class Models with 1-6 Classes

Classes	-Loglikelihood	AIC	BIC	aBIC	Entropy	LRT(*p*)
1	−36990.65	74011.29	74108.59	74060.92	–	–
2	−34102.73	68267.47	68468.55	68370.04	0.77	5733.59 (<0.001)
3	−33705.72	67505.45	67810.30	67660.96	0.73	788.22 (<0.001)
4	−33319.74	66765.47	67174.11	66973.92	0.70	766.33 (<0.001)
5	−33224.66	66607.33	67119.75	66868.71	0.72	188.75 (0.07)
6	−33160.46	66510.93	67127.13	66825.25	0.66	127.46 (0.73)

**Fig. 1:**
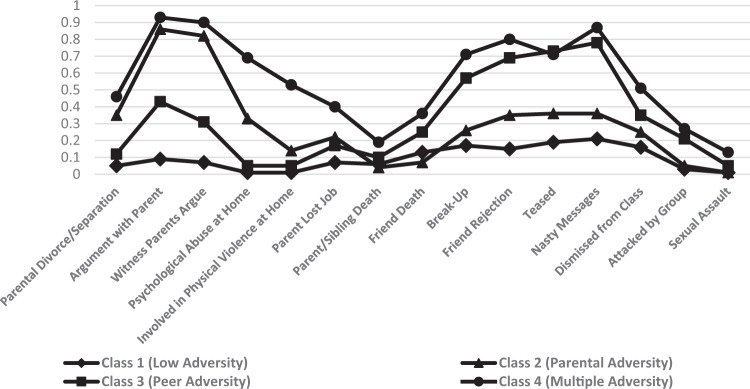
Latent profile plot showing the probability of experiencing each adversity indicator across each of the four observed classes

Class assignment was determined based on participants’ raw posterior probabilities. Results indicated that approximately 42% of the sample (*n* = 2043) had a high probability of belonging to Class 1. Class 1 was the largest class observed and was labelled the Low-Adversity category, as participants in this group showed low probabilities of experiencing any form of adversity. Class 2 consisted of approximately 20% (*n* = 972) of the sample and was characterised by a high probability of experiencing adversity at home. Specifically, participants in this group had a high probability of having serious arguments with their parents and witnessing parental conflict. This class was labelled the Parental-Adversity category. Class 3 comprised approximately 25% (*n* = 1189) of the sample and was labelled the Peer-Adversity category. Participants in this group were likely to experience adversity within peer and friend settings only (e.g. been rejected by friends; been teased by a group). Class 4 was the smallest class detected, consisting of approximately 13% (*n* = 644) of the sample. This class was characterised by a high probability of experiencing adversity across multiple (home, peer and school) settings and was labelled the Multiple-Adversity group. A latent class profile plot of these four classes is displayed in Fig. 1. The average latent class probabilities ranged from 0.76–0.90 for each of the four classes.

The socioeconomic characteristics associated with each class are displayed in Table 4. The multiple adversity class was composed of a high percentage of girls (58%) and those reporting some attraction to the same sex (48%).

**Table 4:** Socio-Demographic Characteristics by Number (%) of Participants in each Latent Class

	Total Sample	Class 1 Low Adversity	Class 2 Parental Adversity	Class 3 Peer Adversity	Class 4 Multiple Adversity
*Gender*					
Male	2404(50%)	1141(56%)	457(47%)	535(45%)	271(42%)
Female	2417(50%)	894(44%)	509(53%)	646(55%)	368(58%)
*Sexuality*					
No Attraction to Same Sex	3090(64%)	1430(70%)	581(60%)	746(63%)	333(52%)
Some Attraction to Same Sex	1758(36%)	615(30%)	391(40%)	443 (37%)	309(48%)
*Place of Birth*					
Ireland	4078(85%)	1775(87%)	781(81%)	1002(85%)	520(81%)
Other	745(15%)	259(13%)	186(19%)	181(15%)	119(19%)
*Age*					
13–15 years	1447(30%)	600(30%)	291(30%)	372(31%)	184(29%)
16–18 years	3377(70%)	1429(70%)	677(70%)	816(69%)	455(71%)
*Mother Education*					
Secondary Level or Lower	1354(34%)	577(34%)	247(31%)	334(34%)	196(38%)
Technical College	475(12%)	188(11%)	102(13%)	131(13%)	54(10%)
University Degree	2189(55%)	948(55%)	456(56%)	517(23%)	268(52%)

### Association between Class Membership, Self-Harm & Suicidality

#### Preliminary Analyses

A series of chi-square analyses were conducted to examine the relationship between most-likely class membership and self-harm thoughts, self-harm behaviours, suicidal thoughts and suicide attempts. Results indicated that there was a significant difference between the classes for self-harm thoughts (χ^2^ (3) = 764.58, *p* < *0.001*, Φ = 0.40), self-harm behaviours (χ^2^ (3) = 659.03, *p* < *0.001*, Φ = 0.37), suicidal thoughts (χ^2^ (3) = 482.16, *p* < *0.001*, Φ = *0.32*), and suicide attempts (χ^2^ (3) = 255.52, *p* < *0.001*, Φ = *0.23*), after adjusting for the family-wise error rate. A detailed overview of the number and percentage of participants in each class experiencing self-harm or suicidality is displayed in Table 5. As can be seen in this table, 70% of adolescents in the multiple adversity group reported having self-harmed and 16% reported attempting suicide within the last six months. In comparison, 17% of adolescents in the low adversity group had self-harmed, and 1% had attempted suicide in the previous six months.

**Table 5:** Number (Percentage) of Participants in Each Class Endorsing Self-Harm & Suicidality Outcomes

	Self-Harm Thoughts		Engaged in Self-Harm		Suicidal Thoughts		Attempted Suicide	
	Yes	No	Yes	No	Yes	No	Yes	No
Class 1 *(Low Adversity)*	521 (26%)	1479 (74%)	336 (17%)	1663 (83%)	190 (9%)	1819 (91%)	21 (1%)	1968 (99%)
Class 2 *(Parental Adversity)*	497 (52%)	468 (48%)	328 (34%)	638 (66%)	188 (19%)	782 (81%)	31 (3%)	938 (97%)
Class 3 *(Peer Adversity)*	732 (62%)	452 (38%)	498 (42%)	686 (58%)	303 (26%)	874 (81%)	58 (5%)	1124 (95%)
Class 4 *(Multiple Adversity)*	519 (81%)	120 (19%)	443 (70%)	194 (30%)	310 (49%)	326 (51%)	100 (16%)	535 (84%)

Spearman’s rho correlations were also carried out to examine preliminary associations between socio-demographic variables (age, gender, sexual orientation), self-harm/suicidality outcomes, and the adversity classes. As can be seen in Table 6, results indicated that while age and gender were significantly and positively associated with self-harm/suicide thoughts and behaviours, few significant age related associations were observed. Therefore, age was not included as a covariate in any further analyses.

**Table 6:** Spearman’s Rho Correlations between all the Adversity Classes, Covariates, Moderators and Self-harm and Suicidality Outcomes

	Age	Sexual Orientation (Same Sex Attaction)	Gender (Female)	Self Harm Thoughts	Self Harm Behaviour	Suicidal Thoughts	Suicide Attempts	Teacher Relations	Home Safety	School Safety	Parental Support	Friend Support	Parental Adversity	Peer Adversity
Sexual Orientation	0.028													
Gender	−0.051^**^	0.081^**^												
Self Harm Thoughts	−0.028	0.156^**^	0.177^**^											
Self Harm Behaviour	−0.011	0.152^**^	0.131^**^	0.679^**^										
Suicidal Thoughts	0.040^*^	0.128^**^	0.112^**^	0.367^**^	0.417^**^									
Suicide Attempts	0.015	0.044^**^	0.103^**^	0.271^**^	0.342^**^	0.578^**^								
Teacher Relations	−0.008	0.004	0.184^**^	−0.092^**^	−0.115^**^	−0.098^**^	−0.113^**^							
Home Safety	−0.012	−0.094^**^	−0.054^**^	−0.229^**^	−0.250^**^	−0.263^**^	−0.232^**^	0.135^**^						
School Safety	0.007	−0.092^**^	0.023	−0.243^**^	−0.235^**^	−0.236^**^	−0.192^**^	0.213^**^	0.345^**^					
Parental Support	0.308	−0.095^**^	0.082^**^	−0.281^**^	−0.276^**^	−0.258^**^	−0.208^**^	0.221^**^	0.386^**^	0.292^**^				
Friend Support	−0.038^*^	−0.012	0.299^**^	−0.075^**^	−0.073^**^	−0.053^**^	−0.039^**^	0.139^**^	0.119^**^	0.268^**^	0.310^**^			
Parental Adversity	−0.008	0.041^**^	0.026	0.041^**^	0.004	−0.011	−0.026	0.022	−0.048^**^	0.017	−0.091^**^	−0.018		
Peer Adversity	−0.016	0.012	0.052^**^	0.165^**^	0.102^**^	0.057^**^	0.033^*^	−0.079^**^	0.029^*^	−0.106^**^	0.002	−0.053^**^	−0.285^**^	
Multiple Adversity	0.021	0.097^**^	0.059^**^	0.266^**^	0.300^**^	0.291^**^	0.255^**^	−0.139^**^	−0.295^**^	−0.193^**^	−0.262^**^	−0.049^**^	−0.196^**^	−0.223^**^

Adversity classes are dummy coded, where each labelled category is coded as 1 and all other groups are coded as 0

^*^p < 0.05, ^**^p < 0.001

#### Moderated Logistic Regression Models

Moderated logistic regression analyses were carried out in order to examine whether perceived home safety, parental support, school safety, teacher relationships, and friend support moderated the relationship between class membership and adolescent self-harm thoughts, self-harm behaviour, suicidal thoughts, and suicide attempts, after controlling for sexual orientation and gender effects. As the observed four-class LCA model was found to have an entropy level less than 0.80, participants’ raw posterior class probability scores were used in each model instead of the “most-likely” nominal class assignment variable, in order to control for any possible classification error that may have resulted from low entropy (Kamata et al., 2018). For each model, the low adversity group was used as the referent category. Perceived home safety, parental support, school safety, teacher relationships, and friend support were simultaneously entered as moderators, along with all interaction terms between each latent class and moderator variables. In order to control for inflation in Type I error due to the number of comparisons being conducted, a Bonferroni correction was applied and a more stringent alpha level of *p* < 0.01 was set for these analyses.

Findings indicated that the predictive model explained between 29–39% of the variance in the outcome measures (suicide thoughts [R^2^ = 0.31, *p* < 0.001], suicide attempts [R^2^ = 0.39, *p* < 0.001], self-harm thoughts [R^2^ = 0.33, *p* < 0.001], and self-harm behaviour [R^2^ = 0.29, *p* < 0.001]. All models were just-identified, and therefore, model fit statistics are not reported. Main and interaction effects for each suicide and self-harm outcome are displayed in Table 7 below. For each model, a significant effect for gender was observed, indicating that females were more likely to experience self-harm and suicide related thoughts and behaviours, compared to males. Results indicated that, after controlling for gender effects, youth adversity experiences were significantly related to their suicidality and self-harm outcomes. Specifically, higher probabilities of experiencing parental (OR = 2.54, CI = 2.17–2.97), peer (OR = 6.40, CI = 5.34–7.66) or multiple adversity (OR = 11.86, CI = 9.10–15.46) were significantly associated with greater likelihood of experiencing self-harm thoughts, and engaging in self-harm behaviours (OR = 1.91, CI = 1.58–2.30; OR = 4.03, CI = 3.46–4.71; OR = 11.07, CI = 8.63–14.19, respectively). Higher probabilities of experiencing peer adversity (OR = 3.29, CI = 2.58–4.20) and multiple forms of adversity (OR = 6.56, CI = 4.95–8.69) were also associated with a higher chance of experiencing recent suicidal thoughts and attempting suicide (OR = 5.38, CI = 2.86–10.14; OR = 12.89, CI = 6.65–25.00, respectively). However, there was no significant association between parental adversity and suicide-related outcomes. Results from the moderated logistic regressions also showed that higher levels of parental support were associated with lower risk of self-harm thoughts (OR = 0.60, CI = 0.52–0.69), self-harm behaviours (OR = 0.58, CI = 0.51–0.67), and suicidal thoughts (OR = 0.58, CI = 0.46–0.72). Additionally, higher levels of perceived safety at school were associated with lower engagement in self-harm behaviours (OR = 0.76, CI = 0.65–0.90) and lower likelihood of experiencing suicidal thoughts (OR = 0.58, CI = 0.51–0.67). However, neither parental support nor school safety were significantly associated with engagement in suicide attempts, after applying the Bonferroni correction. No significant main effects were observed for friend support, teacher relations or perceived safety at home. Furthermore, no significant interactions were observed for any of the suicidality or self-harm outcomes (see Table 7). Parameter estimates and model results when interactions are not included in the model are reported in Appendix A. No sensitivity analyses were performed.

**Table 7:** Unstandardised Estimates (B), Standard Errors (SE), Odds Ratios (OR), and 95% Confidence Intervals (CI) for Each Model

Outcome	Predictors	B	SE	*p*	OR	95% CI OR
Self-Harm Thoughts	***Sexual Orientation***	***0.39***	***0.08***	***<0.001***	***1.47***	***1.26; 1.72***
	***Gender***	***0.83***	***0.09***	***<0.001***	***2.28***	***1.93; 2.70***
	***Parental Adversity***	***0.90***	***0.10***	***<0.001***	***2.46***	***2.04; 2.96***
	***Peer Adversity***	***1.84***	***0.11***	***<0.001***	***6.29***	***5.06; 7.82***
	***Multiple Adversity***	***2.41***	***0.16***	***<0.001***	***11.18***	***8.16; 15.33***
	Teacher Relations	−0.06	0.07	0.43	0.945	0.82; 1.09
	***Parental Support***	***−0.51***	***0.09***	***<0.001***	***0.60***	***0.51; 0.72***
	Friend Support	−0.08	0.08	0.32	0.92	0.78; 1.09
	Home Safety	−0.10	0.10	0.31	0.91	0.75; 1.10
	School Safety	−0.18	0.09	0.04	0.83	0.70; 0.99
	Parental Adversity x Home Safety	−0.03	0.14	0.82	0.97	0.74; 1.28
	Peer Adversity x Home Safety	0.06	0.15	0.69	1.06	0.79; 1.43
	Multiple Adversity x Home Safety	0.03	0.17	0.85	1.03	0.74; 1.44
	Parental Adversity x School Safety	−0.05	0.12	0.67	0.95	0.76; 1.20
	Peer Adversity x School Safety	−0.05	0.12	0.67	0.95	0.76; 1.20
	Multiple Adversity x School Safety	−0.05	0.15	0.75	0.95	0.70; 1.29
	Parental Adversity x Teacher Relations	0.01	0.12	0.93	1.01	0.79; 1.29
	Peer Adversity x Teacher Relations	0.21	0.10	0.04	1.24	1.02; 1.50
	Multiple Adversity x Teacher Relations	−0.08	0.13	0.53	0.92	0.71; 1.19
	Parental Adversity x Parental Support	0.25	0.14	0.08	1.28	0.97; 1.68
	Peer Adversity x Parental Support	−0.03	0.13	0.84	0.97	0.75; 1.26
	Multiple Adversity x Parental Support	0.23	0.17	0.17	1.25	0.91; 1.74
	Multiple Adversity x Parental Support	0.26	0.14	0.06	1.30	0.99; 1.71
	Parental Adversity x Friend Support	−0.07	0.12	0.55	0.93	0.73; 1.18
	Peer Adversity x Friend Support	0.18	0.14	0.21	1.20	0.90; 1.59
Self-Harm Behaviours	***Sexual Orientation***	***0.41***	***0.11***	***<0.001***	***1.51***	***1.23;*** ***1.86***
	***Gender***	***0.60***	***0.09***	***<0.001***	***1.83***	***1.53; 2.18***
	***Parental Adversity***	***0.61***	***0.11***	***<0.001***	***1.83***	***1.47; 2.29***
	***Peer Adversity***	***1.37***	***0.10***	***<0.001***	***3.95***	***3.27; 4.78***
	***Multiple Adversity***	***2.34***	***0.15***	***<0.001***	***10.40***	***7.73; 14.01***
	Teacher Relations	−0.07	0.07	0.33	0.93	0.81; 1.07
	***Parental Support***	***−0.53***	***0.08***	***<0.001***	***0.59***	***0.50; 0.69***
	Friend Support	−0.01	0.10	0.91	0.99	0.81; 1.20
	Home Safety	0.07	0.10	0.52	1.07	0.87; 1.31
	***School Safety***	***−0.26***	***0.10***	***0.009***	***0.77***	***0.63; 0.94***
	Parental Adversity x Home Safety	−0.27	0.17	0.11	0.77	0.55; 1.06
	Peer Adversity x Home Safety	−0.19	0.16	0.25	0.83	0.60; 1.14
	Multiple Adversity x Home Safety	−0.04	0.15	0.81	0.96	0.72; 1.29
	Parental Adversity x School Safety	0.05	0.13	0.69	1.05	0.82; 1.35
	Peer Adversity x School Safety	0.05	0.13	0.69	1.05	0.82; 1.35
	Multiple Adversity x School Safety	0.14	0.13	0.29	1.15	0.89; 1.49
	Parental Adversity x Teacher Relations	−0.09	0.11	0.41	0.92	0.74; 1.13
	Peer Adversity x Teacher Relations	0.15	0.13	0.19	1.16	0.93; 1.45
	Multiple Adversity x Teacher Relations	−0.01	0.13	0.98	1.00	0.77; 1.29
	Parental Adversity x Parental Support	0.27	0.12	0.03	1.32	1.02; 1.70
	Peer Adversity x Parental Support	0.17	0.12	0.14	1.19	0.95; 1.49
	Multiple Adversity x Parental Support	0.12	0.14	0.39	1.13	0.86; 1.50
	Multiple Adversity x Parental Support	0.07	0.15	0.62	1.08	0.81; 1.44
	Parental Adversity x Friend Support	−0.05	0.13	0.74	0.96	0.74; 1.24
	Peer Adversity x Friend Support	−0.01	0.13	0.93	0.99	0.80; 1.29
Suicidal Thoughts	***Sexual*** ***Orientation***	***0.37***	***0.08***	***<0.001***	***1.44***	***1.23;*** ***1.70***
	***Gender***	***0.53***	***0.10***	***<0.001***	***1.70***	***1.39; 2.07***
	Parental Adversity	0.31	0.18	0.09	1.36	0.95; 1.95
	***Peer Adversity***	***1.17***	***0.15***	***<0.001***	***3.22***	***2.42; 4.30***
	***Multiple Adversity***	***1.82***	***0.18***	***<0.001***	***6.16***	***4.37; 8.67***
	Teacher Relations	−0.14	0.10	0.18	0.87	0.71; 1.07
	***Parental Support***	***−0.54***	***0.14***	***<0.001***	***0.58***	***0.45; 0.76***
	Friend Support	−0.10	0.11	0.39	0.91	0.73; 1.13
	Home Safety	−0.13	0.13	0.30	0.88	0.68; 1.13
	***School Safety***	−***0.43***	***0.11***	***<0.001***	***0.65***	***0.53; 0.80***
	Parental Adversity x Home Safety	−0.06	0.17	0.72	0.94	0.68; 1.31
	Peer Adversity x Home Safety	−0.04	0.17	0.82	0.96	0.70; 1.33
	Multiple Adversity x Home Safety	−0.04	0.14	0.78	0.96	0.72; 1.28
	Parental Adversity x School Safety	0.15	0.14	0.29	1.16	0.88; 1.52
	Peer Adversity x School Safety	0.15	0.14	0.29	1.16	0.88; 1.52
	Multiple Adversity x School Safety	0.21	0.14	0.14	1.23	0.94; 1.61
	Parental Adversity x Teacher Relations	0.01	0.16	0.94	1.01	0.74; 1.39
	Peer Adversity x Teacher Relations	0.19	0.15	0.19	1.21	0.91; 1.62
	Multiple Adversity x Teacher Relations	0.10	0.13	0.44	1.01	0.86; 1.41
	Parental Adversity x Parental Support	−0.11	0.20	0.57	0.89	0.60; 1.32
	Peer Adversity x Parental Support	0.14	0.20	0.46	1.16	0.79; 1.70
	Multiple Adversity x Parental Support	0.20	0.16	0.22	1.22	0.89; 1.65
	Multiple Adversity x Parental Support	0.09	0.15	0.58	1.09	0.81; 1.48
	Parental Adversity x Friend Support	−0.12	0.16	0.46	0.89	0.65; 1.22
	Peer Adversity x Friend Support	0.16	0.15	0.29	1.18	0.87; 1.59
Suicide Attempts	Sexual Orientation	−0.06	0.15	0.67	0.94	0.70; 1.27
	***Gender***	***0.78***	***0.18***	***<0.001***	***2.19***	***1.55; 3.09***
	Parental Adversity	0.49	0.42	0.25	1.63	0.71; 3.74
	***Peer Adversity***	***1.69***	***0.39***	***<0.001***	***5.41***	***2.53; 11.55***
	***Multiple Adversity***	***2.57***	***0.41***	***<0.001***	***13.06***	***5.85; 29.13***
	Teacher Relations	−0.42	0.25	0.09	0.66	0.41; 1.06
	Parental Support	−0.60	0.28	0.04	0.55	0.32; 0.96
	Friend Support	−0.01	0.41	0.98	0.99	0.44; 2.21
	Home Safety	−0.22	0.18	0.24	0.81	0.57; 1.16
	School Safety	−0.34	0.28	0.22	0.71	0.41; 1.23
	Parental Adversity x Home Safety	−0.15	0.22	0.48	0.86	0.56; 1.32
	Peer Adversity x Home Safety	−0.03	0.26	0.92	0.98	0.58; 1.64
	Multiple Adversity x Home Safety	0.04	0.20	0.86	1.04	0.70; 1.54
	Parental Adversity x School Safety	0.04	0.30	0.91	1.04	0.58; 1.85
	Peer Adversity x School Safety	0.04	0.30	0.91	1.04	0.58; 1.85
	Multiple Adversity x School Safety	0.01	0.30	0.97	1.01	0.56; 1.84
	Parental Adversity x Teacher Relations	0.42	0.35	0.24	1.52	0.76; 3.04
	Peer Adversity x Teacher Relations	0.13	0.33	0.68	1.14	0.60; 2.17
	Multiple Adversity x Teacher Relations	0.40	0.26	0.13	1.49	0.89; 2.48
	Parental Adversity x Parental Support	0.01	0.41	0.99	1.01	0.45; 2.23
	Peer Adversity x Parental Support	0.38	0.37	0.30	1.47	0.71; 3.06
	Multiple Adversity x Parental Support	0.28	0.30	0.34	1.32	0.74; 2.36
	Multiple Adversity x Parental Support	0.22	0.48	0.64	1.25	0.49; 3.17
	Parental Adversity x Friend Support	0.02	0.47	0.96	1.02	0.41; 2.57
	Peer Adversity x Friend Support	−0.05	0.41	0.91	0.96	0.42; 2.15

Significant associations are highlighted in bold

## Discussion

Although previous research has indicated that experiencing adverse or negative life events is linked with increased risk of suicidality/self-harm (Aytur et al., 2022; Li et al., 2021), less is known about the association between different patterns of adversity and youth suicidality or self-harm, and the protective factors that can help promote resilience among those most at-risk (Gallagher & Miller, 2018). By using a person centred approach to identify the dominant patterns of adversity experienced by adolescents across home, peer and school settings, the findings from the current research extend our understanding about the risk and protective factors relating to youth suicidality and self-harm and provide relevant implications for research and practice.

### Youth Adversity Profiles

The current research identified four dominant profiles of youth adversity, based on adolescents’ negative life experiences across home, peer and school settings; Low adversity; Parental adversity; Peer adversity; and Multiple adversity. While findings indicated that a large number of adolescents (42%) had a low probability of experiencing any form of adversity, the majority (58%) of participants had moderate-high probabilities of experiencing adversity at home with their parents (Parental adversity), with their friends/peers (Peer adversity), or across peer, school and home settings (Multiple adversity). The rates of adversity observed here are comparable to trends reported in other international studies (Broekhof et al., 2022; Flaherty et al., 2013), and suggest that experiences of adversity are prevalent among adolescents in Ireland. It should also be noted that almost half of the multiple-adversity group (48%) were comprised of young people who identified as having some attraction to the same sex. This aligns with findings from previous research which has indicated that LGBTQ + youth may experience elevated levels of adversity (Craig et al., 2020). Given the array of research linking childhood adversity to poorer (short and longer-term) health outcomes (Hughes et al., 2019; Oh et al., 2018), these findings have relevant implications for policy and practice. However, while this research is beneficial and generates further insight into youth’s patterns of adversity and the types of adverse experiences that co-occur, further information regarding the timing, frequency and duration of these adverse experiences, and how these may differ for LGBTQ+ youth, is still needed to help better inform prevention efforts (Lacey & Minnis, 2020; Xiao et al., 2022). This may be an important avenue for future research to explore.

#### The Association between Youth Adversity Profiles and Suicidality & Self-harm

This research provides notable insights into the patterns of adversity experienced by young people in Ireland, and their association with suicidality/self-harm. Similar to findings reported in previous studies (Bruffaerts et al., 2010; McLafferty et al., 2018), the current study found evidence to suggest that youth who are exposed to adversity are more likely to experience suicidal/self-harm thoughts, and engage in suicidal/self-harm behaviours, compared to youth at low risk of adversity. Most notably, the results indicate that youth who likely experience adversity across multiple (e.g. peer, home & school) domains are most at risk of experiencing suicidality and self-harm outcomes. While other research has suggested that exposure to multiple risk factors can be associated with greater maladjustment in children and adolescents (Göbel & Cohrdes, 2021), the current research provides additional insight into the magnitude of this relationship. The large effect sizes observed for the multiple adversity indicator across all suicidality and self-harm outcomes are noteworthy, and have important applied implications as they suggest that youth who experience adversity across multiple domains should be priority targets for intervention.

Furthermore, given the number of young people reporting adverse experiences across multiple domains, and the strength of the association between these experiences and youth suicidality and self-harm, this research also provides support for broadening the definition of adversity to encompass negative experiences that occur across a broad array of youth social ecologies (Lacey & Minnis, 2020). This aligns with research (Lopez et al., 2021; Smyth & Darmody, 2021) and theory (Bronfenbrenner, 1979; Vaughn & Dejonckheere, 2021) from the broader child development literature which contends that youth development is impacted by the interplay between multiple social systems. However, it should be noted that while this research assessed incidents of adversity across a number of social domains, community-level adversity indicators were omitted from this research. Future studies may benefit from including additional indicators that assess adversity across home, peer, school and community levels.

Although the current research did not find evidence of a significant association between parental adversity and suicidality, overall, the findings suggest that the context in which adversity occurs is important, and may have varying associations with adolescent suicidality/self-harm outcomes. The ability to identify subgroups at high risk of suicidality and self-harm has been noted as a priority policy objective (Bilsen, 2018; HSE, 2020), with researchers and practitioners contending that target and/or context specific prevention strategies may be needed in order to reduce risk of suicidality and self-harm among these vulnerable groups (Zalsman et al., 2016). Thus, by helping to identify which subgroups of adolescents may be at increased risk of suicidality and/or self-harm, the current research has notable implications for policy and practice, as these findings may help inform the development of enhanced screening efforts or more targeted intervention and prevention initiatives.

#### The Buffering Effects of Positive Teacher Relations, Social Support and Sense of Safety

While teacher relations, friend support, and feeling safe at home were not found to be associated with self-harm or suicidality outcomes in the current study, findings revealed that higher levels of parental support and perceptions of safety at school were linked to lower risk of youth self-harm behaviours and suicidal thoughts. However, none of the ecological resources examined here were significantly associated with youth engagement in recent suicide attempts. Additionally, none of these supportive factors were found to significantly moderate the relationship between youth’s adversity profiles and suicidality or self-harm outcomes. Researchers refer to variables that reduce negative outcomes at elevated levels of risk as *protective factors*, and distinguish these from *compensatory factors*, which reduce negative outcomes across all risk levels (Wright, Masten & Narayan, 2013; Zimmerman 2013). It is frequently cited in the literature that a key aim of prevention research is to identify the protective factors that can help bolster resilience and promote positive adaptation under conditions of elevated risk (Forster et al., 2020; McLaughlin, 2018). Crucially, the findings from the current research suggest that parental support and perceptions of safety at school act as compensatory factors for youth suicide related thoughts and self-harm, but do not appear to exert a protective effect. This finding contrasts with previous reports which provided evidence to indicate that social support and sense of safety act as both compensatory and protective factors (Gallagher & Miller, 2018; Racine et al., 2020; Taliaferro & Muehlenkamp, 2017). However, much of the evidence in relation to protective factors has emerged from studies employing variable-centred approaches, such as the cumulative risk approach, or those employing more narrow definitions (e.g. family dysfunction, neglect or abuse) of adversity (Forster et al., 2020). These findings therefore highlight the need for further research exploring the protective factors that can buffer or mitigate risk for youth who are exposed to different patterns of adversity. It is also recommended that future research explore the buffering effects of other supportive resources, as the lack of moderating effects observed in the current study may suggest that more targeted supports or resources may be needed in order to mitigate risk among vulnerable groups.

### Implications for Policy & Practice

There are notable implications for policy and practice arising from the findings of this study. First, data indicated that there was a high prevalence of self-harm and suicidality among adolescents. Approximately 33% of young people reported engaging in self-harm, while 4% reported having attempted suicide in the previous six months. Although the levels of self-harm reported here are somewhat higher than those reported in other cross-sectional research with community samples of Irish adolescents (Dooley et al., 2019; Morey, Corcoran, Arensman & Perry, 2008), they align with evidence emerging from the National Registry of Self-Harm (Griffin et al., 2018) which report growing rates of self-harm among young people in Ireland. Thus, these findings provide compelling evidence to suggest that greater youth intervention and prevention work in this area is needed. Additionally, the current research helps address an identified policy need by providing further insight into the characteristics of youth who are likely to experience suicidality/self-harm thoughts and behaviours. These types of insights are crucial for enhancing screening efforts and informing the development of targeted, youth-specific intervention and prevention programmes. One of the aims of the Irish Connecting for Life suicide prevention strategy (HSE, 2020) is to develop targeted approaches that can reduce self-harm and suicide among priority groups, and improve mental health in these individuals. Young people are identified as a priority group in this strategy. Similarly, the WHO Comprehensive Mental Health Action Plan (2013–2030) also identify youth as a key priority group, for whom more comprehensive prevention strategies need to be developed and implemented. By helping to shed light on the sub-groups of youth who are at elevated risk of suicidality and self-harm the findings of this research can help inform policy and guide future prevention efforts.

Moreover, the socio-ecological approach employed in the current research also provides notable insights for policy and practice. Specifically, this research extends understanding of how risk and protective factors from across different social domains interact to influence youth suicidality and self-harm. Given the growing international policy emphasis placed on the importance of developing effective, community-focused suicide/self-harm prevention strategies (e.g., Sharing the Vision; WHO Comprehensive Mental Health Action Plan), this type of research is crucial for helping to inform the development of a multi-contextual response to youth suicide/self-harm. For example, the Irish Connecting for Life policy (HSE, 2020) endorses community-based gatekeeper training as an effective form of suicide prevention (Rochford et al., 2021). Hence, by providing more insight into the social experiences and ecological resources that are associated with increased or decreased youth risk, the current findings can help inform such training initiatives. The finding that parental support and perceived safety at school are associated with lower risk of self-harm and suicide thoughts may be especially relevant for prevention strategies or initiatives which target key gatekeepers, such as parents and schools. In particular, these findings highlight the compensatory role that parental support and safety at school play in reducing risk of self-harm and suicidal thoughts, and suggest these types of ecological resources are important to consider when designing intervention and prevention strategies, as they may produce more potent effects, than other resources, such as peer support. While identifying the factors that can buffer adversity is an important objective, identifying the ecological resources or sources of support that lower youth’s risk of self-harm behaviours and suicide related thoughts generally, regardless of their adversity profiles, is also beneficial as this information can help guide more generic prevention efforts. Nonetheless, the lack of significant interaction effects observed across all outcomes is notable, and indicates that further work is needed to understand the factors that can protect or buffer against adversity.

### Limitations and Directions for Future Research

There are a number of limitations associated with this research. In particular, it should be acknowledged that the cross-sectional nature of this research limits the ability to make inferences about cause-and-effect and future research would benefit by exploring longitudinal associations. Additionally, several of the variables assessed in the current research were measured using single item indicators. While the use of single item indicators is common in the adversity and self-harm literature (Nock et al., 2008), there are limitations associated with this form of measurement (e.g., it may be more susceptible to measurement bias), which should be acknowledged (Millner et al., 2015). It may be beneficial for future research to employ validated scale assessments where possible. Furthermore, although the novel application of a person-centered approach to exploring the association between adversity and youth suicidality and self-harm in this study is a relevant highlight, the current research employed an exploratory LCA framework. In order to provide further support for the four adversity profiles identified in this research, additional confirmatory LCA studies are needed. Relatedly, it is necessary to acknowledge that in the current research adversity was assessed as a yes or no binary experience and the frequency and severity of the adverse experience were not assessed. This is an important limitation as risk may be arbitrarily applied for some participants (Lacey & Minnis, 2020). Further research assessing the frequency, timing and duration of these adverse experiences is needed.

## Conclusion

A plethora of research has indicated that exposure to adversity is a notable risk factor for youth engagement in suicidal and self-harm behaviours, but more research examining whether the patterns of adversity youth experience across multiple social contexts are linked to their endorsement of suicidality/self-harm outcomes is needed. Additionally, there is a lack of understanding about the ecological supports and resources that can help buffer the relationship between adversity and youth suicide/self-harm, which is needed to help strengthen policy and practice efforts. The current research aimed to address these knowledge gaps, with findings indicating that adolescents’ suicidality and self-harm are linked to their experiences of adversity across peer, school, and family contexts. Notably, results suggest that youth who experience adversity across multiple contexts are at elevated risk of suicide and self-harm, and may be important targets for intervention/prevention initiatives. Findings from the current research also suggest that while parental support and feeling safe at school are linked to lower risk of suicidal thoughts and self-harm, these ecological resources do not buffer against adversity. Overall, the findings from the current research highlight the importance of examining the interaction between risk and protective factors across multiple social contexts, but further research is needed to identify the ecological supports and resources that can best protect those at elevated risk levels.

## Supplementary Information

Supplementary Information
