# Supplementary material for: Youth Suicide and Self-Harm: Latent Class Profiles of Adversity and the Moderating Roles of Perceived Support and Sense of Safety
Source: J Youth Adolesc. 2023 Mar 24;52(6):1255–71. doi: 10.1007/s10964-023-01762-1 (PMC10121538; doi:10.1007/s10964-023-01762-1)
Supplement: Supplementary file 1 — Supplementary Information [file 10964_2023_1762_MOESM1_ESM.docx]

**Appendix A**

**Table showing findings from the logistic regression models with main effects only**

|  |  | **Model Fit** | | **Parameter Estimates** | | | | |
| --- | --- | --- | --- | --- | --- | --- | --- | --- |
| **Outcome** | **Predictors** | **R^2^** | ***p*** | **B** | **SE** | ***p*** | **OR** | **95% CI OR** |
| Self-Harm Thoughts | ***Sexual Orientation*** | ***0.33*** | ***<.001*** | ***0.40*** | ***0.08*** | ***<.001*** | ***1.50*** | ***1.29; 1.74*** |
|  | ***Gender*** |  |  | ***0.82*** | ***0.08*** | ***<.001*** | ***2.28*** | ***1.93; 2.69*** |
|  | ***Parental Adversity*** |  |  | ***0.91*** | ***0.09*** | ***<.001*** | ***2.48*** | ***2.09; 2.95*** |
|  | ***Peer Adversity*** |  |  | ***1.85*** | ***0.10*** | ***<.001*** | ***6.37*** | ***5.21; 7.79*** |
|  | ***Multiple Adversity*** |  |  | ***2.40*** | ***0.13*** | ***<.001*** | ***11.06*** | ***8.58; 14.26*** |
|  | Teacher Relations |  |  | -0.00 | 0.04 | 0.93 | 1.00 | 0.92; 1.08 |
|  | ***Parental Support*** |  |  | ***-0.13*** | ***0.02*** | ***<.001*** | ***0.88*** | ***0.86; 0.91*** |
|  | Friend Support |  |  | -0.01 | 0.01 | 0.47 | 0.99 | 0.97; 1.02 |
|  | Home Safety |  |  | -0.12 | 0.09 | 0.18 | 0.89 | 0.74; 1.06 |
|  | ***School Safety*** |  |  | ***-0.25*** | ***0.05*** | ***<.001*** | ***0.78*** | ***0.70; 0.86*** |
| Self-Harm Behaviours | ***Sexual Orientation*** | ***0.29*** | ***<.001*** | ***0.43*** | ***0.11*** | ***<.001*** | ***1.53*** | ***1.25; 1.88*** |
|  | ***Gender*** |  |  | ***0.60*** | ***0.09*** | ***<.001*** | ***1.83*** | ***1.53; 2.19*** |
|  | ***Parental Adversity*** |  |  | ***0.60*** | ***0.11*** | ***<.001*** | ***1.81*** | ***1.47; 2.24*** |
|  | ***Peer Adversity*** |  |  | ***1.37*** | ***0.10*** | ***<.001*** | ***3.94*** | ***3.24; 4.80*** |
|  | ***Multiple Adversity*** |  |  | ***2.29*** | ***0.13*** | ***<.001*** | ***9.91*** | ***7.76; 12.65*** |
|  | Teacher Relations |  |  | -0.05 | 0.04 | 0.18 | 0.95 | 0.88; 1.02 |
|  | ***Parental Support*** |  |  | ***-0.12*** | ***0.01*** | ***<.001*** | ***0.89*** | ***0.87; 0.91*** |
|  | Friend Support |  |  | -0.01 | 0.01 | 0.70 | 1.00 | 0.97; 1.02 |
|  | Home Safety |  |  | -0.09 | 0.08 | 0.23 | 0.91 | 0.79; 1.06 |
|  | ***School Safety*** |  |  | ***-0.24*** | ***0.04*** | ***<.001*** | ***0.79*** | ***0.72; 0.86*** |
| Suicidal Thoughts | ***Sexual Orientation*** | ***0.30*** | ***<.001*** | ***0.38*** | ***0.08*** | ***<.001*** | ***1.46*** | ***1.25; 1.71*** |
|  | ***Gender*** |  |  | ***0.52*** | ***0.10*** | ***<.001*** | ***1.69*** | ***1.39; 2.05*** |
|  | ***Parental Adversity*** |  |  | ***0.39*** | ***0.15*** | ***0.01*** | ***1.48*** | ***1.10; 2.00*** |
|  | ***Peer Adversity*** |  |  | ***1.16*** | ***0.15*** | ***<.001*** | ***3.19*** | ***2.39; 4.25*** |
|  | ***Multiple Adversity*** |  |  | ***1.65*** | ***0.15*** | ***<.001*** | ***5.19*** | ***3.85; 6.99*** |
|  | Teacher Relations |  |  | -0.05 | 0.04 | 0.26 | 0.96 | 0.88; 1.04 |
|  | ***Parental Support*** |  |  | ***-0.15*** | ***0.02*** | ***<.001*** | ***0.86*** | ***0.84; 0.89*** |
|  | Friend Support |  |  | -0.03 | 0.01 | 0.08 | 0.98 | 0.95; 1.00 |
|  | ***Home Safety*** |  |  | ***-0.30*** | ***0.09*** | ***0.001*** | ***0.74*** | ***0.63; 0.89*** |
|  | ***School Safety*** |  |  | -0.34 | 0.05 | <.001 | 0.71 | 0.65; 0.78 |
| Suicide Attempts | Sexual Orientation | ***0.34*** | ***<.001*** | -0.05 | 0.16 | 0.75 | 0.95 | 0.70; 1.29 |
|  | ***Gender*** |  |  | ***0.75*** | ***0.17*** | ***<.001*** | ***2.12*** | ***1.51; 2.96*** |
|  | Parental Adversity |  |  | 0.65 | 0.33 | 0.05 | 1.91 | 1.01; 3.62 |
|  | ***Peer Adversity*** |  |  | ***1.51*** | ***0.37*** | ***<.001*** | ***4.55*** | ***2.19; 9.44*** |
|  | ***Multiple Adversity*** |  |  | ***2.22*** | ***0.33*** | ***<.001*** | ***9.24*** | ***4.88; 17.49*** |
|  | Teacher Relations |  |  | -0.13 | 0.07 | 0.06 | 0.88 | 0.76; 1.01 |
|  | ***Parental Support*** |  |  | ***-0.11*** | ***0.03*** | ***<.001*** | ***0.89*** | ***0.85; 0.94*** |
|  | Friend Support |  |  | -0.01 | 0.03 | 0.97 | 1.00 | 0.95; 1.05 |
|  | Home Safety |  |  | -0.41 | 0.09 | ***<.001*** | 0.66 | 0.55; 0.80 |
|  | School Safety |  |  | -0.37 | 0.08 | ***<.001*** | 0.69 | 0.59; 0.80 |

*Note: Significant associations are highlighted in bold.*
